# Isolated skyrmion, skyrmion lattice and antiskyrmion lattice creation through magnetization reversal in Co/Pd nanostructure

**DOI:** 10.1038/s41598-021-98337-6

**Published:** 2021-09-23

**Authors:** Sateesh Kandukuri, V. Satya Narayana Murthy, P. K. Thiruvikraman

**Affiliations:** Department of Physics, BITS Pilani Hyderabad Campus, Jawahar Nagar, Kapra Mandal, Medchal District, Hyderabad, 500078 Telangana India

**Keywords:** Engineering, Electrical and electronic engineering, Materials science, Electronic devices, Information storage, Nanoscience and technology, Nanoscale devices, Nanoscale materials, Physics, Ferromagnetism, Magnetic properties and materials, Spintronics, Topological matter

## Abstract

Skyrmion and antiskyrmion spin textures are axisymmetric inhomogeneous localized objects with distinct chirality in magnetic systems. These spin textures are potential candidates for the next generation energy-efficient spintronic applications due to their unique topological properties. Controlled and effective creation of the spin textures is required to use in conventional and neuromorphic computing applications. Here we show by micromagnetic simulations creating an isolated skyrmion, skyrmion lattice and antiskyrmion lattice through the magnetization reversal in Co/Pd multilayer nanostructure using spin-polarized current. The spin textures' stability depends on the spin-polarized current density, current pulse width, and Dzyaloshinskii–Moriya interaction (DMI). Antiskyrmions are evolved during the formation of a single skyrmion and skyrmion lattice. Skyrmion and antiskyrmion lattices together are observed for lower pulse width, 0.05 ns. Our micromagnetic studies suggest that the two distinct lattice phases' evolution could help to design the topological spin textures-based devices.

## Introduction

From 1989, theoretical studies predicted the existence of chiral spin textures in magnetic materials with broken inversion symmetry^1–4^. In 2009, skyrmions were first observed as a hexagonal lattice in a chiral magnet MnSi^5^ due to the presence of the bulk Dzyaloshinskii–Moriya interaction (DMI)^6,7^. Subsequently, magnetic skyrmions were found in similar non-centrosymmetric B20 compounds^8,9^ and also in multi-layered structures (stacking of ferromagnet and heavy metal)^10–13^ with interfacial DMI^14,15^. Chiral skyrmion's non-trivial topology explains their stability from untwisting into a saturated state (a uniformly polarized state). So, the skyrmions are treated as axisymmetric inhomogeneous localized finite particles with a well-defined chirality between their core (± m_z_) and the periphery ($\mp$ m_z_). There is a difference in the chirality of the skyrmions observed in the bulk compounds (Bloch skyrmions—spins rotate in the plane perpendicular to the propagation direction) and thin films (Neel skyrmions—spins rotate along the propagation direction) due to the type of DMI.

The stable topological spin textures are characterized by an invariant integer topological charge defined as Q = 1/4π ∫d^2^**r**·**m**(**r**)·[∂_x_·**m**(**r**) x ∂_y_·**m**(**r**)], where **m**(**r**) is the direction of the magnetic moment at **r** = (r cos φ, r sin φ)^16–18^. The topological charge, also called skyrmion number or winding number, indicates the number of times the reduced magnetization wraps the unit sphere. Skyrmion like axisymmetric spin textures are parametrized as **m**(**r**) = [cos **Φ**(**r**) sin **Θ**(**r**), sin **Φ**(**r**) sin **Θ**(**r**), cos **Θ**(**r**)] with **Φ**(**r**) = νφ + η. The terms vorticity (ν) and helicity (η) are related to the magnetization rotation from the core to the periphery of the spin texture. Once the boundary conditions (spin state at the core and periphery) are fixed, vorticity determines the topological charge, and helicity determines the type of spin texture. The two possible chiral structures for the Bloch and Neel skyrmions with the spin-up magnetization (+ m_z_) at the core are (Q, ν, η) = (1, 1, ± π/2) (+ π/2 corresponds to clockwise and - π/2 for the counter-clockwise direction of the spins in a plane perpendicular to the radial direction) and (Q, ν, η) = (1, 1, 0 or π) (0 corresponds to the spins divergence and π for convergence in a plane along the radial direction) respectively. The spin texture with centre magnetization + m_z_, a negative topological charge, and negative vorticity is referred to as antiskyrmion (− 1, − 1, η)^16–18^. For an antiskyrmion with |Q| = 1, helicity varies between 0 and 2π—the structure will have a two-fold rotational symmetry with alternative Bloch and Neel type spin rotations.

Recent studies on the nontrivial topological spin texture such as skyrmions, merons, and bimerons^19–22^ are expected to be the key components of next-generation energy-efficient spintronics known as skyrmionics. For the conventional^23–26^, neuromorphic^27,28^, and reservoir computing^29^ applications, controlled and effective creation of spin textures are required. Until recently, these spin textures have been created by reversing the magnetization at the system's desired location. In 2020, a review article by Xichao Zhang et al.^18^ discussed the creation of a magnetic skyrmion using an external magnetic field, spin-polarized current, local electric field, laser, and imprinting. In 2014, Wataru Koshibae and Naoto Nagaosa showed skyrmions and antiskyrmions by local heating^30^. Antiskyrmions are observed in bulk materials with lower symmetry^1,31^ and later are experimentally observed at room temperature in Heusler compounds with D_2d_ symmetry^32^. Hoffmann^33^ and Güngördü^34^ have predicted that interfacial DMI with C_2v_ symmetry in ultrathin films can form antiskyrmions.

There are different methods of creating skyrmions as mentioned in the review article, Ref.^18^, we are focusing on their creation by employing a perpendicularly applied spin-polarized current. In 2012, Youngbin Tchoe and Jung Hoon Han theoretically predicted creating an isolated skyrmion using a perpendicularly applied spin current to a circular chiral ferromagnet^35^. In 2013 Sampaio et al., have numerically studied the nucleation of an isolated skyrmion in a circular disk by sending the spin-polarized current through a nano-contact^36^. In the same year, Romming et al. experimentally created a single isolated skyrmion in Pd/Fe bilayer system by using a spin-polarized scanning tunneling microscope (SP-STM)^37^. In 2016 Yuan and Wang, employing micromagnetic simulations, demonstrated the skyrmion formation in a spin valve nanostructure using a designed nanosecond current pulse^38^. They observed a skyrmion only when the fixed layer magnetization is in the plane and perpendicular to the free layer magnetization. Yin et al. theoretically suggested how to create a single skyrmion in helimagnetic thin films^39^. In 2017, Legrand et al. experimentally created the skyrmions by applying a uniform spin current into nano-tracks^40^. In the same year, Woo et al. experimentally demonstrated the skyrmion creation by applying bipolar spin current pulse into a Pt/CoFeB/MgO multilayer^41^. Hrabec et al. also shown the creation of skyrmion by applying electric current through asymmetric electric contacts placed on symmetric magnetic bilayer system^42^.

In this article, it is being presented the formation of an isolated skyrmion, skyrmion lattice, and antiskyrmion lattice using different spin-polarized current pulses into Co/Pd nanostructure. The above spin texture formation is explained based on the magnetization reversal through spin-transfer torque (STT— a resultant torque due to the interaction between the polarized itinerant electrons and the free layer localized electrons)^43–46^. The reversal behavior depends on the current density, pulse width, and DMI. Asymmetric behavior of STT is observed for lower current densities. The evolution of antiskyrmion lattice is observed by merging adjacent incomplete skyrmions for lower current densities and longer pulse widths. It also has been observed the formation of the skyrmion and antiskyrmion lattice together for higher current densities and lower current pulse widths through dumbbell spin textures (Q = 0).

## Results

### System and simulations

We have considered a square nanostructure consisting of a fixed/spacer/free layer of dimensions 200 nm × 200 nm. The fixed and free layers magnetization is perpendicular to the plane of the nanostructure. The effective thickness of the Co free layer is 3 nm^47^. The spacer layer, Pd, introduces the interfacial DMI required for the formation of magnetic skyrmions.

The dynamics of the free layer are governed by the Landau–Lifshitz equation with the Slonczewski spin-transfer torque^26,36,38,48,49^

1 $$\frac{{d{\mathbf{m}}}}{dt} = - \left| \gamma \right|{\mathbf{m}} \times {\mathbf{H}}_{{{\mathbf{eff}}}} - \frac{\alpha \left| \gamma \right|}{{M_{s} }}{\mathbf{m}} \times \left( {{\mathbf{m}} \times {\mathbf{H}}_{{{\mathbf{eff}}}} } \right) + {{\varvec{\uptau}}}_{{{\mathbf{STT}}}}$$

here **m** is the normalized unit vector of magnetization, M_s_ the saturation magnetization, γ the gyromagnetic ratio, α Gilbert’s damping parameter, and **τ**_STT_ due to the spin-polarized current applied perpendicular to the plane of the nanostructure.

2 $${{\varvec{\uptau}}}_{{{\mathbf{STT}}}} = \beta \frac{{\varepsilon - \alpha \varepsilon^{\prime}}}{{1 + \alpha^{2} }}\left( {{\mathbf{m}} \times \left( {{\mathbf{m}}_{{\mathbf{P}}} \times {\mathbf{m}}} \right)} \right) - \beta \frac{{\varepsilon^{\prime} - \alpha \varepsilon }}{{1 + \alpha^{2} }}\left( {{\mathbf{m}} \times {\mathbf{m}}_{{\mathbf{P}}} } \right)$$

3 $$\beta = \frac{{\hbar j_{Z} }}{{M_{S} et}}$$

4 $$\varepsilon = \frac{{P\Lambda^{2} }}{{\left( {\Lambda^{2} + 1} \right) + \left( {\Lambda^{2} - 1} \right)\left( {{\mathbf{m}} \cdot {\mathbf{m}}_{{\mathbf{P}}} } \right)}}$$

here **m**_**P**_ is the fixed layer magnetization, j_z_ the current density along the z-axis, e the charge of the electron, t the thickness of the free layer, P the spin polarization, Λ the Slonczewski parameter which characterizes the spacer layer and ε′ the secondary spin–torque parameter.

H_eff_ in Eq. (1) can be deduced from the total free energy density, E(m)

5 $$H_{eff} = - \frac{1}{{\mu_{0} M_{S} }}\int {E\left( m \right)} dv$$

where μ_0_ is the permeability of free space and

6 $$E\left( {\mathbf{m}} \right) = A\sum {\left( {\nabla m} \right)}^{2} + D\left[ {m_{z} \nabla \cdot {\mathbf{m}} - \left( {{\mathbf{m}} \cdot \nabla } \right)m_{z} } \right] - K_{u} m_{z}^{2} - \frac{{M_{S} }}{2}\mu_{0} {\mathbf{m}} \cdot {\mathbf{H}}_{{\mathbf{d}}} - M_{s} {\mathbf{m}} \cdot {\mathbf{B}}$$

The first term is the exchange energy with the stiffness constant A, the second term is the interfacial DMI energy with coefficient D, out of plane uniaxial anisotropy gives the third term, dipolar interaction gives the fourth term, and the Oersted field produced by the electrical current gives the fifth term. The competition among the energies is responsible for the final magnetic configuration in the nanostructure. After defining the energies, the simulations have been done using mumax3^48^ with a mesh size of 1 nm × 1 nm × 1 nm. The following magnetic parameters are used in the simulations: M_s_ = 280 kA/m; A = 15 pJ/m; K_u_ = 0.06 MJ/m^3^, α = 0.1 and D = 0.30–0.50 mJ/m^2^^13^. The spin polarization is taken as P = 0.4 and for the symmetrical free and fixed layers Λ = 1, and ε′ = 0^26,36,38,49^.

The skyrmions have been created in the free layer for various electrical current pulse widths, and current densities applied perpendicular to the plane of the nanostructure. The size of the skyrmion is defined as the diameter of the circle of m_z_ = 0 contour.

### Skyrmion formation

#### Current pulse width 1.0 ns

**Figure 1:**
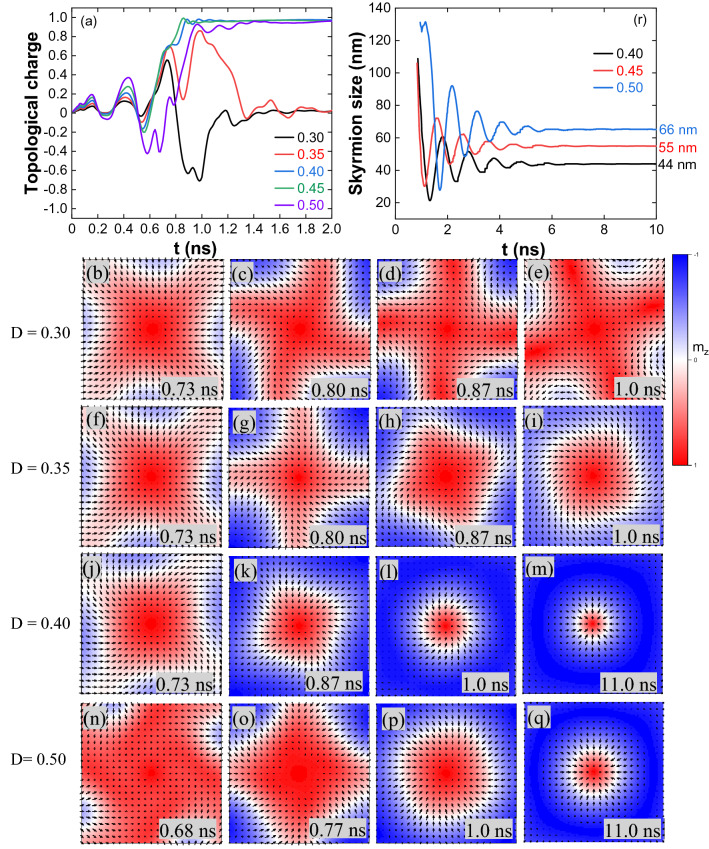
(**a**) Topological charge variation for J = 2 × 10^11^ A/m^2^ and 1.0 ns pulse width. (**b**–**e**) The captured spin states reflect the topological charge variation at different times for D = 0.30 mJ/m^2^, red colour indicates the magnetization along the + z direction, blue along the − z direction and white in the plane. (**f**–**i**) For D = 0.35 mJ/m^2^. (**j**–**m**) For D = 0.40 mJ/m^2^. (**l**) Skyrmion at 1.0 ns and (**m**) skyrmion stabilized after breathing mode. Similar type of states are observed for D = 0.45 mJ/m^2^. (**n**–**q**) For D = 0.50 mJ/m^2^, the size of the stable skyrmion in (**q**) at 11.0 ns is bigger than in (**m**, D = 0.40 mJ/m^2^). (**r**) Skyrmion stabilization through breathing mode and the size of the skyrmion increases with DMI [Mumax3 is used for simulations, Ref.^48^].

The work started with a current density (J) of 1 × 10^11^ A/m^2^ applied perpendicular to the nanostructure for 1.0 ns. The spin-polarized current does not induce the magnetization reversal for all the ranges of DMI. Increasing the current density to 2 × 10^11^ A/m^2^ single skyrmion is observed for D ≥ 0.4 mJ/m^2^. The topological charge variation for J = 2 × 10^11^ A/m^2^ is shown in Fig. 1a. For D = 0.30 mJ/m^2^, from 0.73 ns (Q = 0.6) to 1.0 ns (Q = − 0.7) the magnetization reversal at the edge centre moves in the counter-clockwise direction to the adjacent edge through some intermediate states (Fig. 1b–e). In the relaxation, the spin state is annihilated by reducing its size. For D = 0.35 mJ/m^2^, the reversal behaviour is similar to 0.30 mJ/m^2^ up to 0.87 ns (Fig. 1f,g). The magnetization reversal continued from the edge towards the centre of the nanostructure (Fig. 1h) and created a skyrmion-like spin texture (Q = 0.9, Fig. 1i) at the edge of the pulse. As the periphery of the spin texture is close to the nanostructure's edge, this spin state is annihilated by expanding its size in the relaxation. For D = 0.40 and 0.45 mJ/m^2^, the captured spin states indicate the early reversal of magnetization (Fig. 1j,k). It has created a skyrmion (Q = 1) at the nanostructure centre (Fig. 1l). Once the current pulse is off, the skyrmion is stabilized through breathing mode^50^, as shown in Fig. 1m (Supplementary Movie 1). For D = 0.50 mJ/m^2^, the topological charge variation is similar to lower DMIs up to 0.6 ns, and a skyrmion (Q ≈ 1) is formed at the edge of the pulse through some intermediate states (Fig. 1n–p). And it is stabilized through breathing mode (Fig. 1q). Variation of the skyrmion size with time shown in Fig. 1r. The oscillations indicate the breathing mode, and the skyrmion is stabilized after 5 ns.

**Figure 2:**
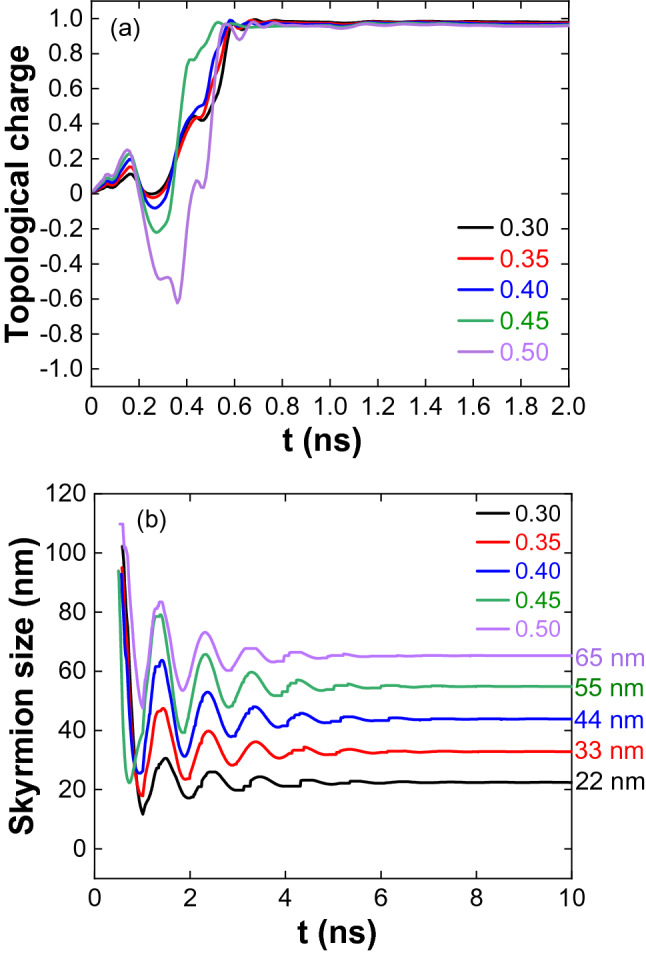
(**a**) Topological charge variation for J = 3 × 10^11^ A/m^2^ and 1.0 ns pulse width. (**b**) Skyrmion stabilization through breathing mode and the size of the skyrmion increases with DMI.

**Figure 3:**
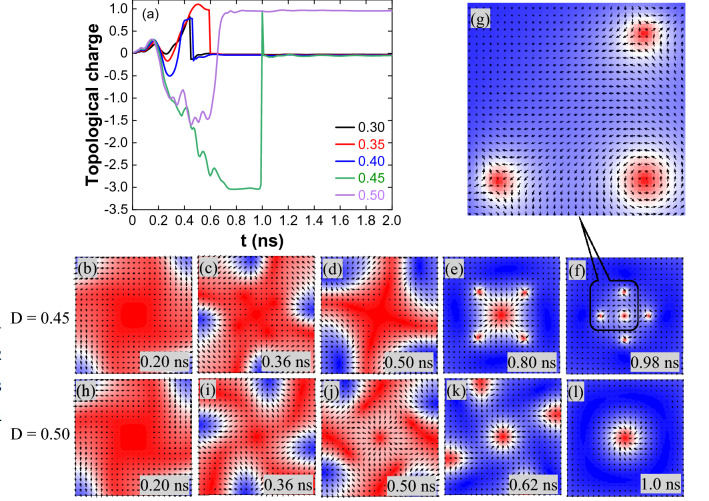
(**a**) Topological charge variation for J = 4 × 10^11^ A/m^2^ and 1.0 ns pulse width. (**b**–**f**) The captured spin states reflect the topological charge variation at different times for D = 0.45 mJ/m^2^, (**e**) at 0.80 ns and (**f**) at 0.98 ns have Q = − 3.0 is because of the centre skyrmion with Q =  + 1 and four antiskyrmions with a total Q = − 4.0. (**g**) The captured enlarged image clearly shows the orientations of the spins, indicating the formation of the centre skyrmion and antiskyrmions. (**h**–**l**) For D = 0.50 mJ/m^2^, (**k**) at 0.62 ns have Q = − 1.5 is due to the centre skyrmion and incomplete antiskyrmions at the edges. (**l**) Centre skyrmion after the annihilation of incomplete antiskyrmions.

Increasing J to 3 × 10^11^ A/m^2^, a stable (1, 1, π) skyrmion is formed for all the DMIs by magnetization reversal from the corners (Fig. 2a,b show the topological charge and skyrmion size variation with time). Figure 3a shows the topological charge variation for J = 4 × 10^11^ A/m^2^. For D = 0.30 and 0.35 mJ/m^2^, magnetization reversal from the edge centres combines to form a skyrmion at the centre and is annihilated by reducing its size. For D = 0.40 mJ/m^2^, skyrmion formed by reversal from the corners, lengthen along the edges and expanded towards the centre. Later it is annihilated by reducing its size (spin states for J = 4 × 10^11^ A/m^2^ and D = 0.30–0.40 mJ/m^2^ are given in Supplementary Fig. S1). For D = 0.45 mJ/m^2^, the reversal from the corners moves in a counter-clockwise direction to the edge centre, as shown in Fig. 3b,c. The spin orientation and the topological charge indicate the incomplete formation of the (− 1, 1, 0) skyrmions at the edges. The reversal extended and moved to the other corner, as shown in Fig. 3d. (− 1, 1, γ) antiskyrmions are evolved by merging the boundaries of the adjacent incomplete skyrmions. The calculated topological charge − 3 for the lattice shows it contains a (1, 1, γ) skyrmion surrounded by four antiskyrmions (Fig. 3e,f). The difference in the chirality of the skyrmion and antiskyrmion can be observed in the enlarged image, Fig. 3g. It is noted that the helicity of the skyrmion and antiskyrmions is changing due to an increase in the field-like torque (spin states are given in Supplementary Fig. S2, Supplementary Movie 2). First, the antiskyrmions are annihilated by reducing their size, followed by the skyrmion annihilation. For D = 0.50 mJ/m^2^, up to 0.5 ns, the reversal process is similar to D = 0.45 mJ/m^2^, creates a skyrmion at the centre and incomplete antiskyrmions at the edges (Fig. 3h–k). The antiskyrmions are annihilated by moving along the edges, and then the skyrmion at the centre (Fig. 3l) is stabilized through breathing mode (Supplementary Movie 3).

**Figure 4:**
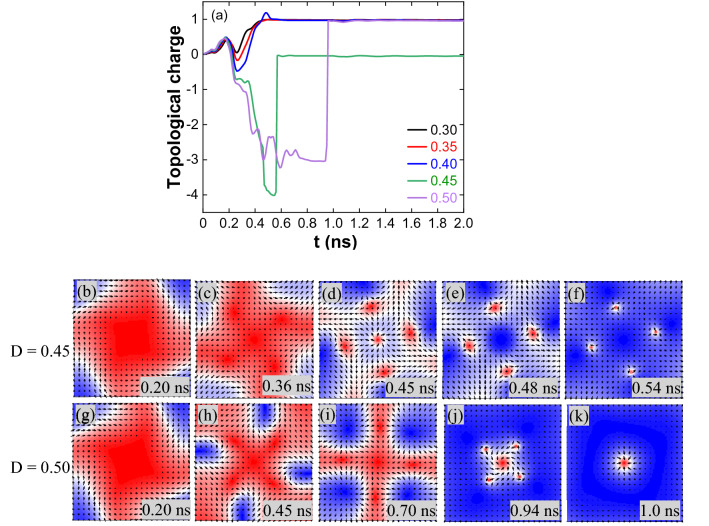
(**a**) Topological charge variation for J = 5 × 10^11^ A/m^2^ and 1.0 ns pulse width. (**b**–**f**) The captured spin states reflect the topological charge variation at different times for D = 0.45 mJ/m^2^, (**d**) at 0.45 ns have Q = -3.0 is because of the centre skyrmion with Q =  + 1 and four antiskyrmions with a total Q = − 4.0. (**e**) At 0.48 ns the centre skyrmion is annihilated and the four antiskyrmions have Q = − 4.0. (**f**) Antiskyrmions size is reduced during the annihilation process. (**g**–**k**) For D = 0.50 mJ/m^2^, (**j**) at 0.94 ns have Q = − 3.0 is due to the centre skyrmion and four antiskyrmions. (**k**) Centre skyrmion after the annihilation of incomplete antiskyrmions.

For J = 5 × 10^11^ A/m^2^, a stable skyrmion is formed for all the DMIs except for 0.45 mJ/m^2^. Figure 4a shows the topological charge variation. The formation of the stable skyrmion is similar for D = 0.30–0.40 mJ/m^2^ (Supplementary Fig. S3). For the D = 0.45 mJ/m^2^, magnetization reversal from the adjacent corners and around the nanostructure centre forms an antiskyrmion lattice surrounding a skyrmion (Fig. 4b–d). First, the skyrmion is annihilated by reducing its size (Fig. 4e) followed by antiskyrmions annihilation (Supplementary Movie 4). For the D = 0.50 mJ/m^2^, magnetization reversal moved from one corner to the adjacent corner in a counter-clockwise direction (Fig. 4f–h). Antiskyrmion lattice surrounding a skyrmion is formed due to combining the magnetization reversal from the adjacent corners (Fig. 4i,j). Later at 0.96 ns, the antiskyrmions are annihilated by reducing their size, and the skyrmion (Fig. 4k) is stabilized through breathing mode (Supplementary Movie 5).

**Figure 5:**
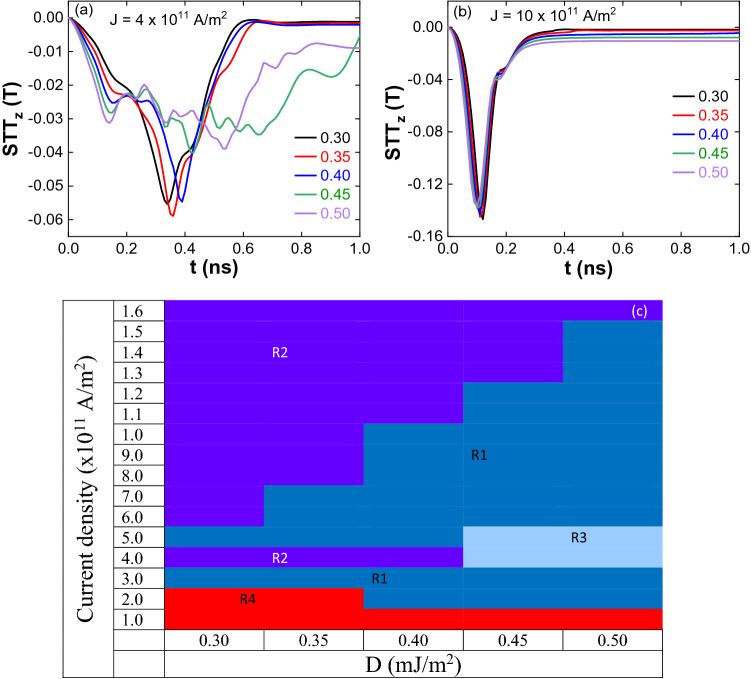
STT variation at different DMIs (**a**) J = 4 × 10^11^ A/m^2^ and (**b**) J = 10 × 10^11^ A/m^2^. STT is more complex and uncomparable for smaller currents (J < 6 × 10^11^ A/m^2^), and for higher currents, systematic variation is observed. (**c**) Phase diagram of stable and unstable skyrmions. Region one (R1) indicates the stable skyrmions—as the DMI increases, the current density range for the formation of the stable skyrmion increases, region two (R2) unstable skyrmions (annihilation by reducing the size), region three (R3) antiskyrmion lattice as the intermediate state during the skyrmion formation and annihilation and in region four (R4) skyrmions have not been observed.

At higher current densities (J ≥ 6 × 10^11^ A/m^2^), a single skyrmion is formed through the magnetization reversal from the corners of the nanostructure for all the values of DMI. The behavior of STT is more complex and un-comparable for lower current densities (J < 6 × 10^11^ A/m^2^, Fig. 5a) to different DMIs, and for higher currents, a systematic variation is observed (Fig. 5b). The formation of the stable and unstable skyrmion at different current densities and DMIs is shown in Fig. 5c. It is observed that as the DMI increases, the current density ranges for the formation of the stable skyrmion increases (region 1). For a given DMI above the threshold current density, the skyrmion is annihilated by reducing its size during the current pulse (region 2). To see the impact of the current pulse width on the stability of the skyrmion, we have reduced the pulse width to 0.5 ns.

#### Current pulse width 0.5 ns

**Figure 6:**
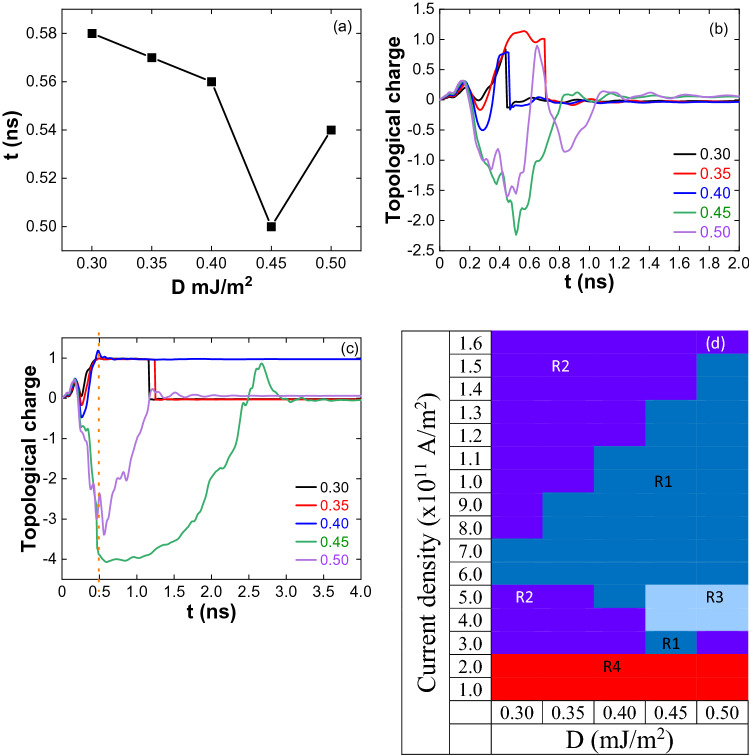
(**a**) Skyrmion formation time dependence on DMI for J = 3 × 10^11^ A/m^2^ and pulse width 1.0 ns. Formation happens on or above 0.5 ns. Topological charge variation for 0.5 ns pulse width (**b**) J = 4 × 10^11^ A/m^2^ and (**c**) J = 5 × 10^11^ A/m^2^. (**d**) Phase diagram of stable and unstable skyrmions for 0.5 ns pulse width. R1 indicates the stable skyrmions, R2 unstable skyrmions (annihilation by reducing the size), R3 antiskyrmion lattice as the intermediate state during the skyrmion formation and annihilation and in R4 skyrmions have not been observed.

In the case of 1.0 ns pulse and for J = 2 × 10^11^ A/m^2^, a skyrmion is formed after 0.5 ns. It is the reason for the non-formation of the skyrmion for 0.5 ns pulse width. Figure 6a shows the nonlinearity in the skyrmion formation time with DMI for 1.0 ns at J = 3 × 10^11^ A/m^2^. Except for D = 0.45 mJ/m^2^, skyrmion formation started during the relaxation, and finally, the skyrmion annihilated for other DMIs. For D = 0.45 mJ/m^2^, skyrmion formed at the end of the pulse (for 0.5 ns) and is stabilized in the relaxation.

Figure 6b shows the topological charge for J = 4 × 10^11^ A/m^2^. Up to D = 0.40 mJ/m^2^, the skyrmion formation, and annihilation process is similar to 1.0 ns pulse. For D = 0.45 mJ/m^2^, the topological charge increases negatively to the edge of the pulse (Q = − 2), and decreasing to zero indicates the formation and annihilation of incomplete skyrmions at the periphery of the nanostructure (Supplementary Movie 6). For D = 0.50 mJ/m^2^, the oscillatory nature in the topological charge is due to the merging of the incomplete skyrmions in the relaxation (Supplementary Movie 7).

Figure 6c is the topological charge for J = 5 × 10^11^ A/m^2^. Skyrmion is annihilated in the relaxation for D = 0.30 and 0.35 mJ/m^2^ and is stable for the D = 0.40 mJ/m^2^. It could be due to overcoming DMI energy than exchange energy for D = 0.40 mJ/m^2^. At D = 0.45 mJ/m^2^ and for 1.0 ns pulse, antiskyrmion lattice is formed below 0.5 ns and is annihilated immediately due to the current. In the present case, since the current is switched off at 0.5 ns, antiskyrmion lattice is existed up to ~ 1.1 ns. Finally, the antiskyrmions are annihilated through the formation of incomplete skyrmions (Supplementary Movie 8). For D = 0.50 mJ/m^2^, Q = − 3 (0.5 ns) to 0 (1.2 ns) indicates the formation and annihilation of incomplete skyrmions at the periphery of the nanostructure (Supplementary Movie 9).

For higher current densities (J ≥ 6 × 10^11^ A/m^2^), the skyrmion formation (< 0.5 ns) and stabilization are the same as like 1.0 ns pulse. Figure 6d shows the skyrmion phase diagram at different current densities and DMIs. By decreasing the pulse width from 1.0 to 0.5 ns, the maximum current density required to form the stable skyrmion has increased. We also observed the stability for a given DMI depends on the pulse width. To confirm this further, we have investigated the skyrmion formation for 0.1 ns pulse.

#### Current pulse width 0.1 ns

**Figure 7:**
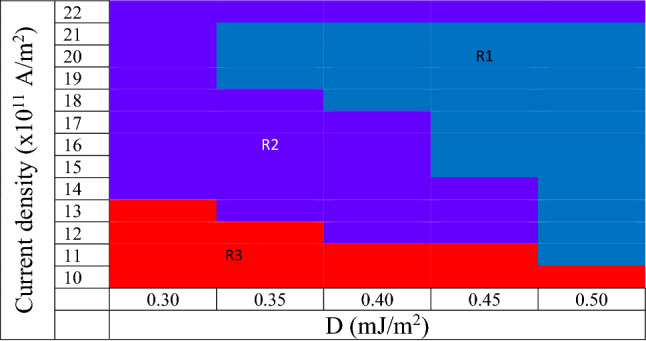
Phase diagram of stable and unstable skyrmions for 0.1 ns pulse width. R1 indicates the stable skyrmions, R2 unstable skyrmions (annihilation by reducing the size) and R3 skyrmions have not been observed. As the DMI increases, the minimum current density required for the formation of the stable skyrmion decreases. The maximum current density at which stable skyrmion observed is 21 × 10^11^ A/m^2^ and is the same for all DMIs.

Skyrmion phase diagram for 0.1 ns pulse width is shown in Fig. 7. It can conclude that as the DMI increases, the minimum current density required for stable skyrmion formation decreases. The maximum current density at which stable skyrmion is observed is 2 × 10^12^ A/m^2^ and is the same for all DMIs. It is also noted for a given DMI, the time at which the nucleation of the skyrmion starts depends on the current density and pulse width. The pulse width has decreased to 0.05 ns to verify the maximum current density dependence on the skyrmion formation time. Interestingly skyrmion lattice has been observed.

### Skyrmion and antiskyrmion lattices

**Figure 8:**
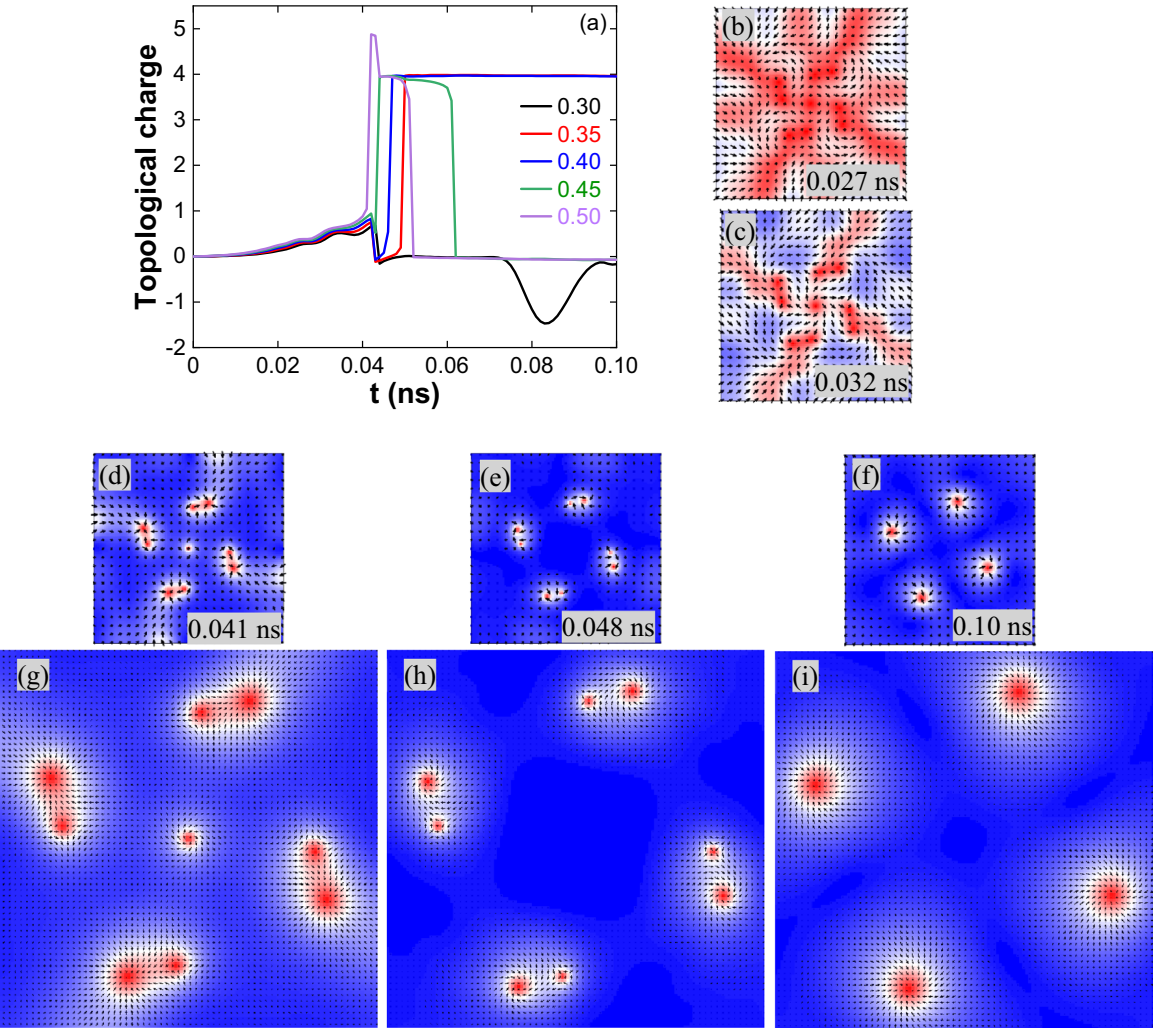
(**a**) Topological charge variation for J = 5 × 10^12^ A/m^2^ and 0.05 ns pulse width. (**b**–**f**) For D = 0.35 mJ/m^2^, spin states observed during the skyrmion lattice formation. (**b**, **c**) Magnetic stripes and (**d**) centre skyrmion surrounded by dumbbell spin texture. The dumbbell spin texture contains a skyrmion and an antiskyrmion with Q = 0. (**e**) Skyrmion and antiskyrmion lattice separation from the dumbbell spin texture, Q = 0 at 0.048 ns. (**f**) Skyrmion lattice after the annihilation of antiskyrmion lattice. (**g**–**i**) are the enlarged images of (**d**–**f**). A similar type of textures is observed for other DMIs also.

The topological charge variation for J = 5 × 10^12^ A/m^2^ and pulse width 0.05 ns is shown in Fig. 8a. The magnetization reversal started from the boundary of the nanostructure. Magnetic stripes are evolved symmetrically (Fig. 8b,c); later, a centre skyrmion surrounded by dumbbell shape spin textures are formed (Fig. 8d). For D = 0.30 mJ/m^2^, the centre skyrmion is annihilated by reducing its size, followed by dumbbell spin textures moving towards the edges. For D = 0.35–0.50 mJ/m^2^, one end of the dumbbell is turned into the skyrmion and the other end into antiskyrmion (Fig. 8e)^51^. The central skyrmion and antiskyrmion lattice are annihilated by reducing their size (Fig. 8e,f). The enlarged images of Fig. 8d–f are shown in Fig. 8g–i, respectively.

**Figure 9:**
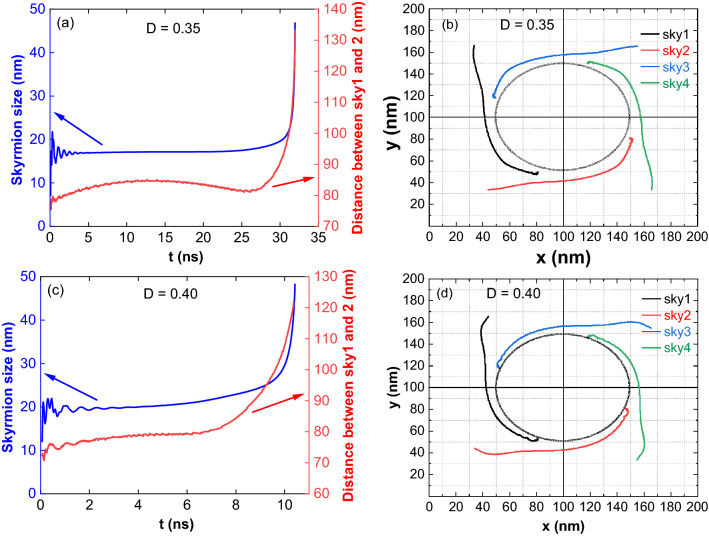
Breathing and gyrotropic modes of skyrmion lattice. (**a**) For D = 0.35 mJ/m^2^ after breathing mode, the skyrmion size is almost constant before annihilation. The separation between the skyrmion 1 and 2 initially increases due to the interaction between them. At maximum separation, repulsion between the skyrmions goes to a minimum, and they behave like isolated and try to come to the centre through the gyrotropic motion. Hence, the separation between the skyrmions decreases. After reaching a minimum separation, the repulsion dominates, and the skyrmions annihilate by moving to the edges. (**b**) Skyrmion movement in the lattice. The reference circle is an eye guide to understand the skyrmion motion. All the four skyrmions have similar behaviour. (**c**) For D = 0.40 mJ/m^2^, the size of skyrmion increases during relaxation leading to an increase in the repulsion and separation. (**d**) Compared to D = 0.35 mJ/m^2^, the skyrmion motion from the reference circle indicates the absence of gyrotropic motion and the domination of skyrmion repulsion because of the bigger size.

After the current pulse is switched off, for D = 0.35 mJ/m^2^, the skyrmions simultaneously showed breathing mode and gyrotropic mode (Fig. 9a)^52,53^. The reference circle in Fig. 9b is a guide to understand the skyrmion motion in the relaxation process. Initially, the separation increases due to the interaction between the skyrmions (Fig. 9a)^54–56^. At the maximum separation, the repulsion between them goes to a minimum, and they behave like isolated skyrmions. (An isolated skyrmion away from the nanostructure centre comes to the centre through the gyrotropic motion in the relaxation^52,53^). Hence, the separation between the skyrmions decreases. Once they come closer, the repulsion dominates, leading to the increase of separation and annihilation at the edges (Supplementary Movie 10).

For D = 0.40 mJ/m^2^, in the relaxation, the size of the skyrmion and the separation between them increases (Fig. 9c,d). An increase in the separation between skyrmions is mainly due to the skyrmion–skyrmion interaction leading to the lattice annihilation at the edges. For D = 0.45–0.50 mJ/m^2^, immediately after the current pulse is switched off, the lattice is annihilated by reducing the skyrmions' size.

**Figure 10:**
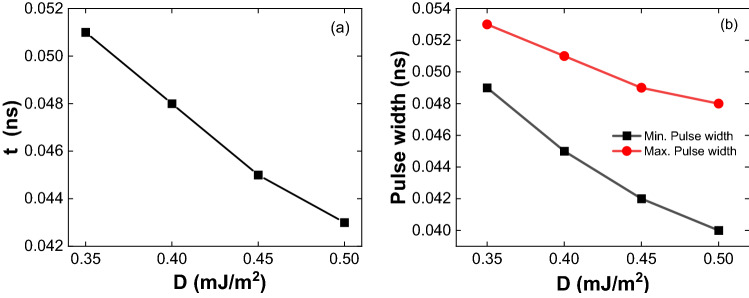
Skyrmion lattice formation time and pulse width dependence on DMI. (**a**) Formation time decreases with an increase in DMI. (**b**) The minimum and maximum pulse widths decrease, and the pulse width range increases with increasing DMI.

Figure 10a,b are the skyrmion lattice formation time and the pulse width dependence with DMIs, respectively. As the DMI increases, the formation time has decreased. The above type of skyrmion lattice formation depends on the pulse width. The minimum and maximum pulse widths decrease, and the pulse width range increases with increasing DMI.

## Conclusions

We have observed magnetization reversal from the corners of the system due to STT forms an isolated skyrmion. Lower current densities required longer pulse width to form a skyrmion, and it is stabilized through breathing mode. For J < 6 × 10^12^ A/m^2^, the action of STT on the free layer is more complex and un-comparable to different DMIs. It could be the reason for the unsystematic formation of the skyrmion and antiskyrmions. By decreasing the pulse width to 0.05 ns and increasing the current density to 5 × 10^12^ A/m^2^, skyrmion and antiskyrmion lattices together are formed from the evolution of the symmetrical magnetic stripes. Antiskyrmion lattice is annihilated during the pulse, and the skyrmion lattice is stable for a longer duration in the case of lower DMIs. Finally, it is predicted a stable skyrmion lattice and antiskyrmion lattice can be created by tuning the material parameters with the current density and pulse width.

## Supplementary Information

Supplementary Information.

Supplementary Video 1.

Supplementary Video 2.

Supplementary Video 3.

Supplementary Video 4.

Supplementary Video 5.

Supplementary Video 6.

Supplementary Video 7.

Supplementary Video 8.

Supplementary Video 9.

Supplementary Video 10.
