# Supplementary material for: Isolated skyrmion, skyrmion lattice and antiskyrmion lattice creation through magnetization reversal in Co/Pd nanostructure
Source: Sci Rep. 2021 Sep 23;11:18945. doi: 10.1038/s41598-021-98337-6 (PMC8460664; doi:10.1038/s41598-021-98337-6)
Supplement: Supplementary file 1 — Supplementary Information. [file 41598_2021_98337_MOESM1_ESM.pdf]

Isolated skyrmion, skyrmion lattice and antiskyrmion lattice creation through magnetization reversal in Co/Pd nanostructure

Supplementary Figures

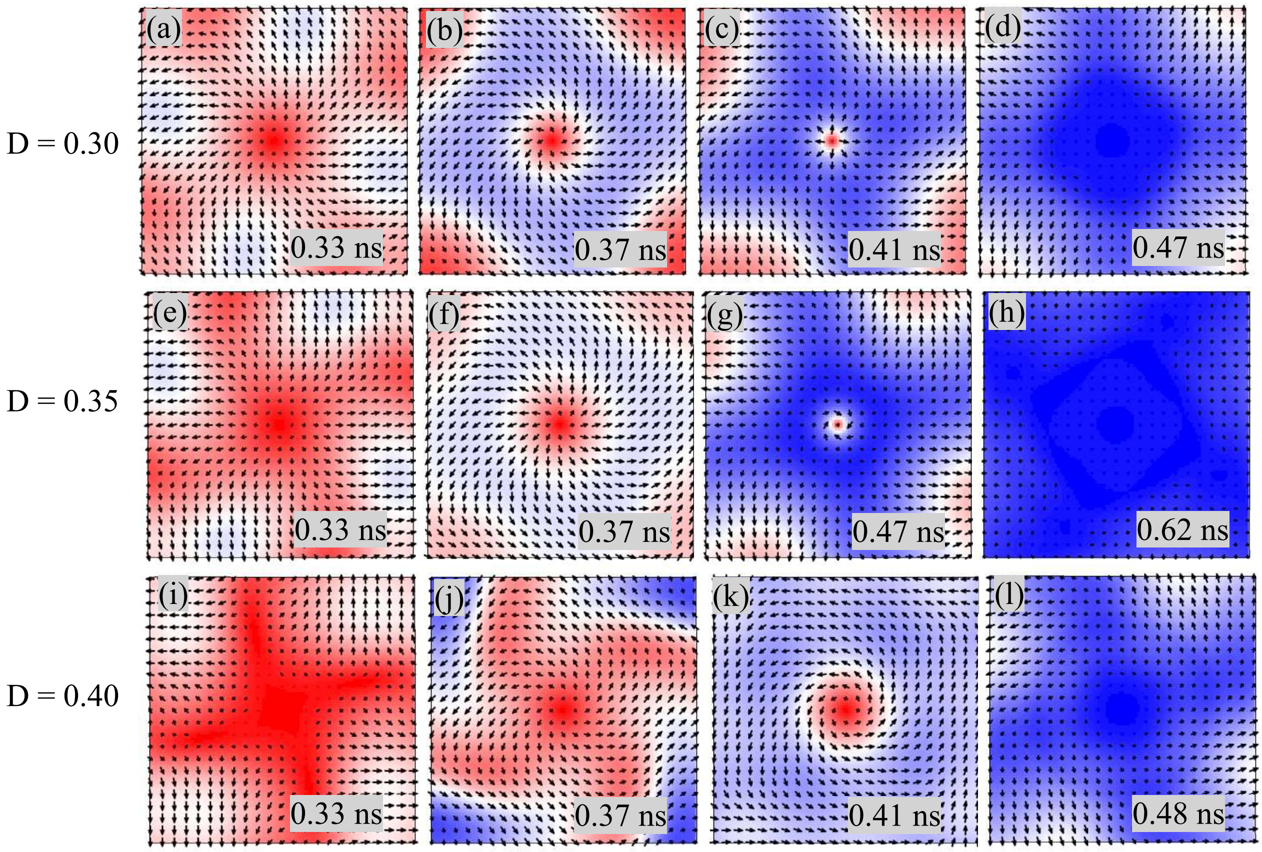

Fig. S1: Captured spin states for J = 4 x 10^{11} A/m^2 and 1.0 ns pulse width. Red colour indicates the magnetization along the +z direction, blue along the –z direction and white in the plane. **(a) – (d)** For D = 0.30 mJ/m^2 and **(e) – (h)** are for D = 0.35 mJ/m^2, the skyrmion formation from the edge centre to annihilation. **(i) – (l)** For D = 0.40 mJ/m^2, skyrmion formation from the corners to annihilation.

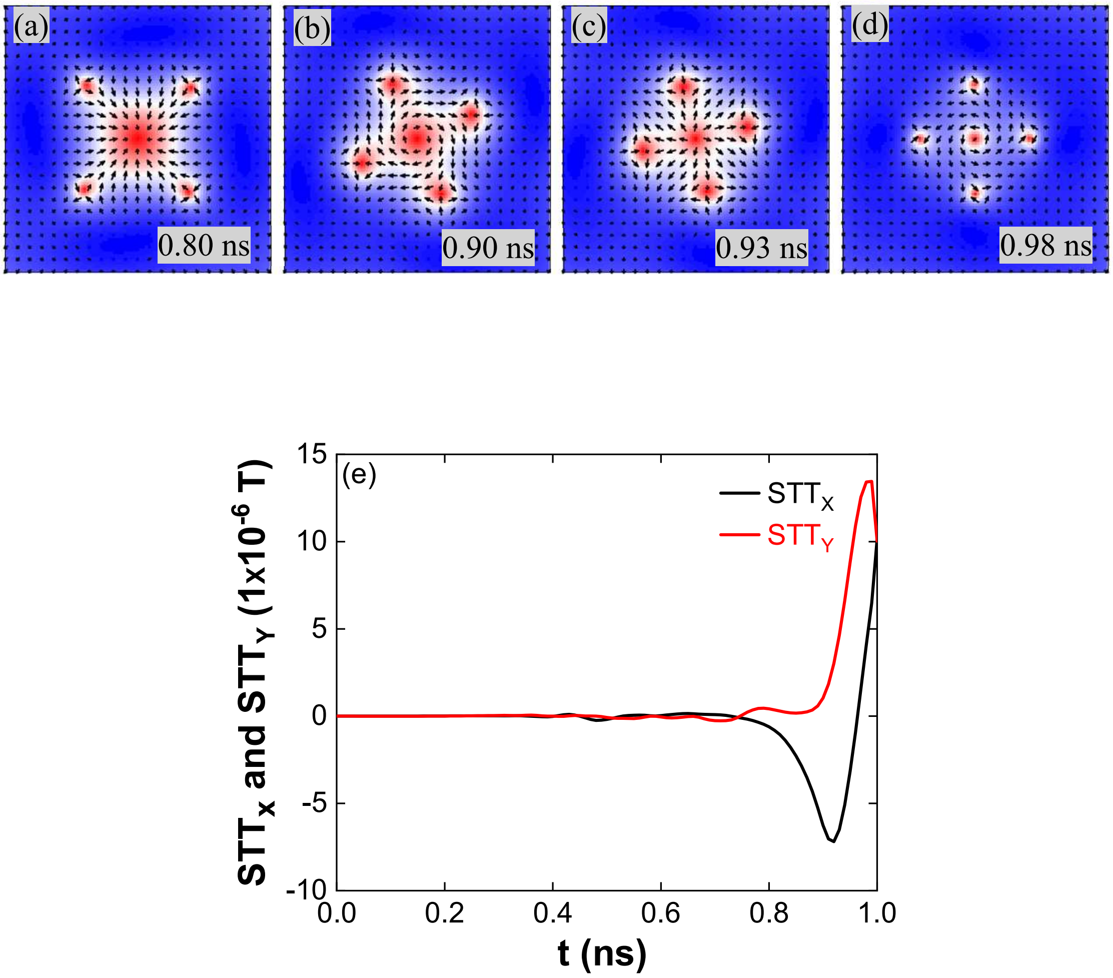

Fig. S2: Helicity of skyrmion and antiskyrmion lattice for J = 4 x 10^{11} A/m^2, 1.0 ns pulse width and D = 0.45 mJ/m^2. **(a)** (1, 1, π) skyrmion, and (-1, -1, -π/2) antiskyrmion. **(b)** (1, 1, -π/2) skyrmion, and (-1, -1, 0) antiskyrmion. (c) (1, 1, 0) skyrmion and (-1, -1, 0) antiskyrmion. **(d)** (1, 1, π/2) skyrmion and (-1, -1, π/2) antiskyrmion. The helicity of the skyrmion and antiskyrmions change is due to the field like torque. (e) Field like torque variation. Large variation in STT is observed after ≈ 0.8 ns.

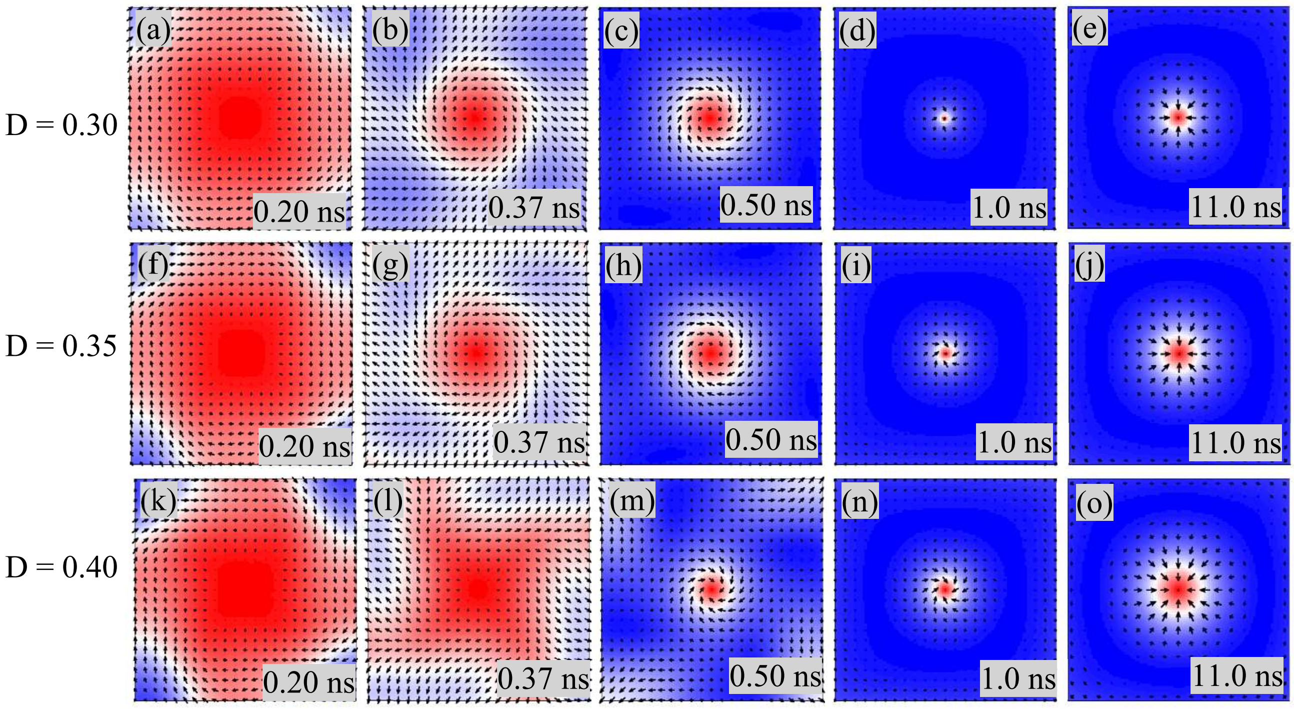

Fig. S3: Formation of stable skyrmion for J = 5 x 10^{11} A/m^2 and 1.0 ns pulse width. **(a) – (e)** For D = 0.30 mJ/m^2, **(f) – (j)** are for D = 0.35 mJ/m^2 and **(k) – (o)** for D = 0.40 mJ/m^2.

Movie 1: Formation of stable skyrmion for J = 2 x 10^{11} A/m^2, 1.0 ns pulse width and D = 0.40 mJ/m^2. The spin states are shown from 0.0 – 1.0 ns with a step time of 0.01 ns and the final stable skyrmion at 11.0 ns.

Movie 2: Formation of skyrmion and antiskyrmion lattice for J = 4 x 10^{11} A/m^2, 1.0 ns pulse width and D = 0.45 mJ/m^2. The spin states are shown from 0.0 – 1.03 ns with a step time of 0.01 ns.

Movie 3: Formation of skyrmion and antiskyrmion lattice for J = 4 x 10^{11} A/m^2, 1.0 ns pulse width and D = 0.50 mJ/m^2. The spin states are shown from 0.0 – 1.0 ns with a step time of 0.01 ns.

Movie 4: Formation of skyrmion and antiskyrmion lattice for J = 5 x 10^{11} A/m^2, 1.0 ns pulse width and D = 0.45 mJ/m^2. The spin states are shown from 0.0 – 0.60 ns with a step time of 0.01 ns.

Movie 5: Formation of skyrmion and antiskyrmion lattice for J = 5 x 10^{11} A/m^2, 1.0 ns pulse width and D = 0.50 mJ/m^2. The spin states are shown from 0.0 – 1.0 ns with a step time of 0.01 ns.

Movie 6: Formation of skyrmion and antiskyrmion lattice for J = 4 x 10^{11} A/m^2, 0.5 ns pulse width and D = 0.45 mJ/m^2. The spin states are shown from 0.0 – 1.0 ns with a step time of 0.01 ns.

Movie 7: Formation of skyrmion and antiskyrmion lattice for J = 4 x 10^{11} A/m^2, 0.5 ns pulse width and D = 0.50 mJ/m^2. The spin states are shown from 0.0 – 1.0 ns with a step time of 0.01 ns.

Movie 8: Formation of skyrmion and antiskyrmion lattice for J = 5 x 10^{11} A/m^2, 0.5 ns pulse width and D = 0.45 mJ/m^2. The spin states are shown from 0.0 – 3.0 ns with a step time of 0.01 ns.

Movie 9: Formation of skyrmion and antiskyrmion lattice for J = 5 x 10^{11} A/m^2, 0.5 ns pulse width and D = 0.50 mJ/m^2. The spin states are shown from 0.0 – 1.30 ns with a step time of 0.01 ns.

Movie 10: Formation of skyrmion lattice and antiskyrmion lattice together for J = 5 x 10^{12} A/m^2, 0.05 ns pulse width and D = 0.35 mJ/m^2. The spin states are shown from 0.0 – 100 ps with a step time of 1.0 ps.
